# ANTI-AMYLOID THERAPIES FOR ALZHEIMER’S DISEASE: PATIENTS’ AND FAMILIES’ PERCEPTIONS

**DOI:** 10.1093/geroni/igae098.2642

**Published:** 2024-12-31

**Authors:** Kaitlyn Lucca, Allison Lapins, Darby Morhardt

**Affiliations:** Northwestern University, Evanston, Illinois, United States; Northwestern University, Evanston, Illinois, United States; Northwestern University, Evanston, Illinois, United States

## Abstract

With new drug therapies available aimed to slow the progression of Alzheimer’s Disease (AD), this study hopes to gain insight into the perceptions, concerns and perceived barriers from patients and families who are impacted by AD. Drug consultations for these therapies are currently taking place at the Northwestern Neurobehavior and Memory Clinic with potentially eligible patients and their families. Study aims are to: 1) measure patient and family perception, barriers and concerns of the drug therapy following a formal consultation or informal discussion with a neurologist, and 2) gather data on the patient and family experience with the drug consultation as a method for making an informed decision. After consultation with their behavioral neurologist, participants are asked to complete the survey with a study team member. To date, 10 participants have completed the survey following consultation with a neurologist. Preliminary results found the highest reported concerns with starting anti-amyloid therapies to be medical complications [larger brain bleeds, brain swelling, micro-hemorrhages] and financial burden. The majority of participants’ interest levels remained the same or were increased following the consultation. Those who reported a decrease in interest level were concerned that the therapy would not be impactful on their daily life. Families will continue to be surveyed following consultation with a neurologist with additional results reported in the coming months.

